# Supplementary material for: Resilience of the Asian atmospheric circulation shown by Paleogene dust provenance
Source: Nat Commun. 2016 Aug 4;7:12390. doi: 10.1038/ncomms12390 (PMC4976207; doi:10.1038/ncomms12390)
Supplement: Supplementary Information — Supplementary Figures 1-3, Supplementary Tables 1-2 and Supplementary References. [file ncomms12390-s1.pdf]

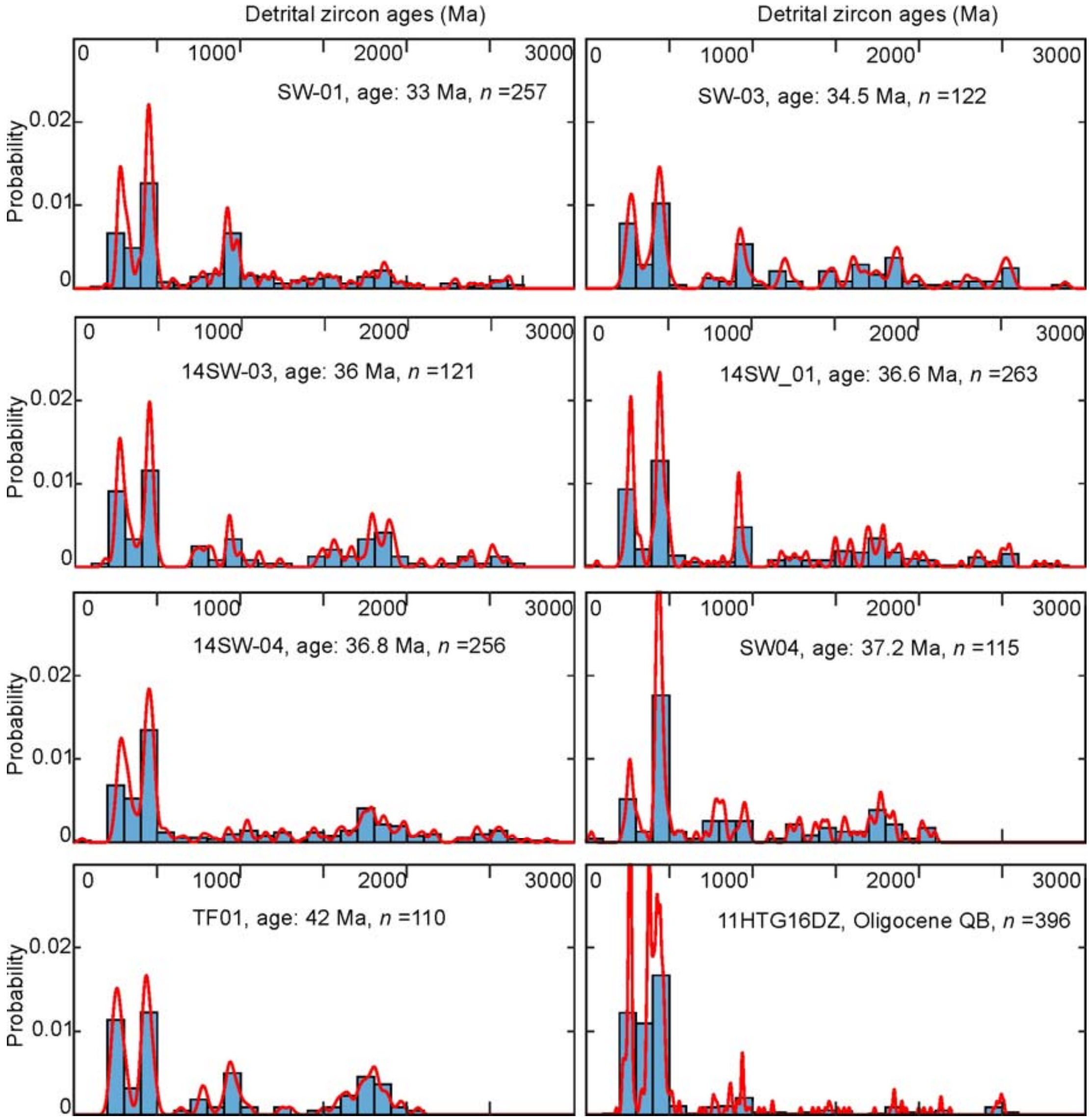

Supplementary Figure 1. ***Kernel density plots (red curves) and histograms (100 Ma bins) of the 8 samples presented in this study****. The kernel density bandwidth of each plot was determined with plug-in bandwidth selection method of Botev et al.[1].*

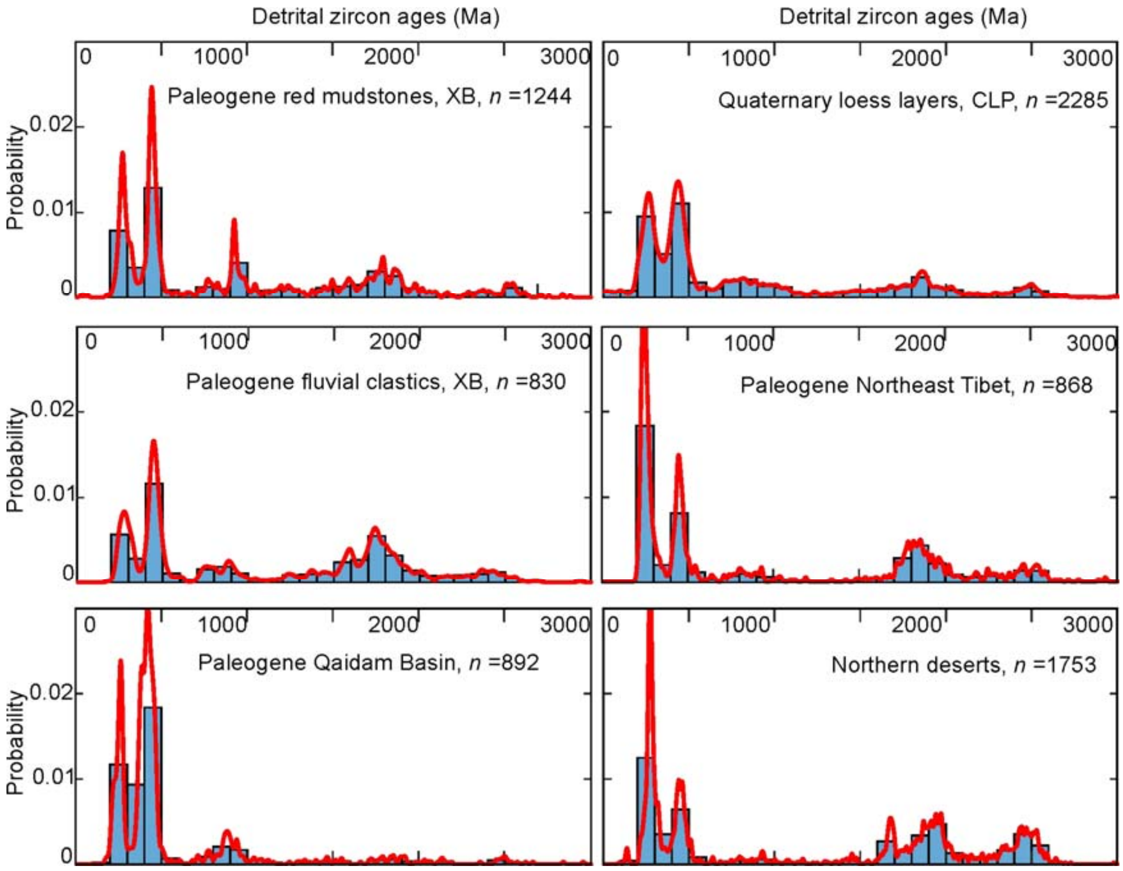

Supplementary Figure 2. ***Probability density plots (red curves) and histograms (100 Ma bins)*** *for* ***the compilations used in the study****: Paleogene red mudstones and fluvial clastics of the Xining Basin (XB), Quaternary loess deposits of the Chinese Loess Plateau (CLP), Paleogene deposits of the Qaidam Basin, Paleogene deposits at the margin of Northeast Tibet, and Quaternary to Mesozoic deposits from the Northern Desert (Mu Us, Badan Jaran and Tengger deserts). Data compilation in Supplementary Data, location of compiled samples in Fig. 1.*

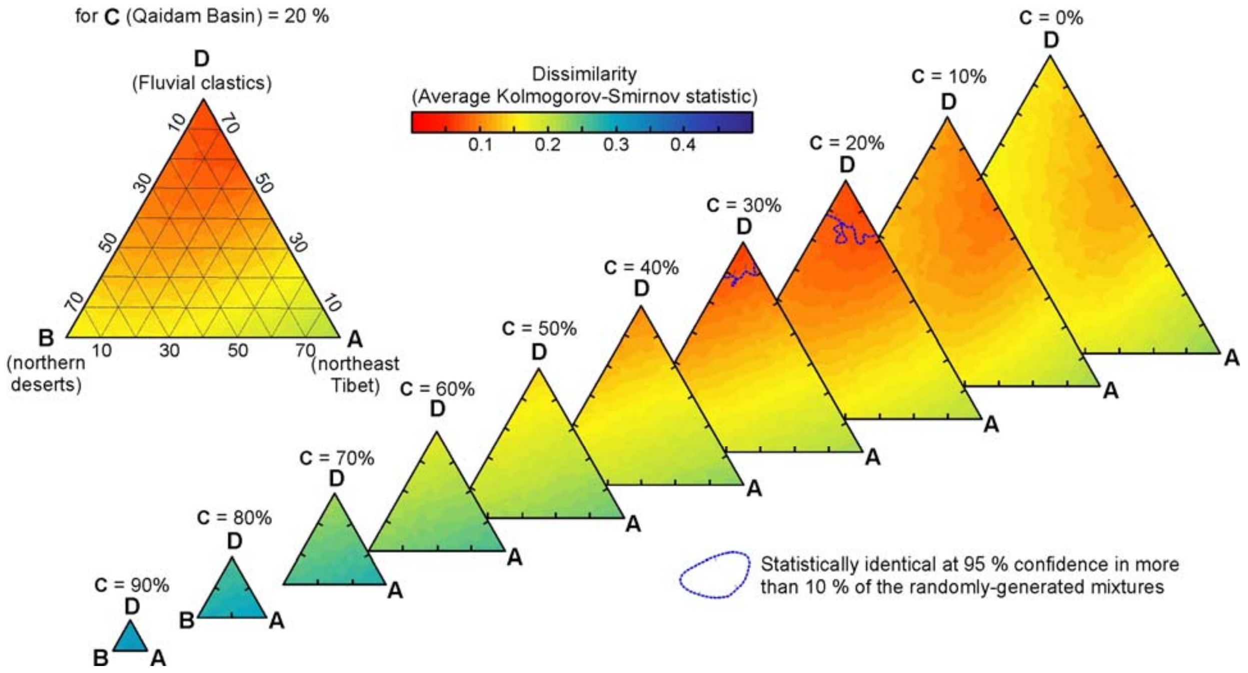

Supplementary Figure 3. ***Dissimilarity to the red mudstones for each possible combination of the four potential dust sources.*** *A: northeast Tibet; B: northern deserts; C: Qaidam Basin; D: Fluvial clastics. Each triangle is a ternary diagram of contribution for the provinces A, C and D for a given contribution of C (contributions of A+B+C+D = 100 % for each triangle; see example for C = 20 % on the left side of the figure). Diagrams are shown every 10 % step of increasing contribution of C for simplification; Note however that we calculated dissimilarities for all the possible mixtures with steps of 2% for every region (A,B,C, and D). The color bar indicates the ranges of values for the dissimilarity measure, here the KS statistic averaged for the N random synthetic distributions per source combination (N=200). The combinations that best fit the red mudstone age distribution are the ones that give the lowest dissimilarity values. An alternative way to determine the best fit is to look at the range of combinations for which more than 10 % of the N random synthetic distributions are statistically similar to the loess (here in the sense of the KS statistic at the 95 % confidence level), highlighted in blue in the figure. This range is limited to Qaidam Basin contributions ranging between 15 to 35 %.*

**XINING BASIN – PALEOGENE RED MUDSTONES**				
**Sample**	**n**	**Nature**	**Coordinates**	**Ref**
SW01	257	Red mudstone, Mahalagou Fm, 33 Ma	36.6604°N, 101.87°W	This paper
SW03	122	Red mudstone, Mahalagou Fm, 34.5 Ma	36.6604°N, 101.87005°W	This paper
14SW03	121	Red mudstone, Mahalagou Fm, 36 Ma	36.6604°N, 101.87°W	This paper
14SW01	263	Red mudstone, Mahalagou Fm, 36.6 Ma	36.6604°N, 101.87°W	This paper
14SW04	256	Red mudstone, Mahalagou Fm, 36.8 Ma	36.6604°N, 101.87°W	This paper
SW04	116	Red mudstone, Mahalagou Fm, 37.2 Ma	36.658531°N, 101.87054°W	This paper
TF01	110	Red mudstone, Mahalagou Fm, 42 Ma	36.575783°N, 101.88044°W	This paper
**XINING BASIN – PALEOGENE FLUVIAL DEPOSITS**				
SW-11-01	94	Fluvial sandstone, Malahagou Fm (40-25.5 Ma)	36.6°N, 101.8°W	2
SW-11-02	98	Fluvial sandstone, Malahagou Fm (40-25.5 Ma)	37.6°N, 101.8°W	2
SW-11-05	92	Fluvial sandstone, Malahagou Fm (40-25.5 Ma)	38.6°N, 101.8°W	2
SW-11-06	98	Fluvial sandstone, Xieja-Chetougou Fm (25.5-18 Ma)	39.6°N, 101.8°W	2
HY-11-01	85	Fluvial sandstone, Upper Cretaceous	36.7°N, 101.04°W	2
HY-11-02	82	Fluvial sandstone, Qijiachuan Fm (50-44 Ma)	37.7°N, 101.04°W	2
HY-11-03	96	Fluvial sandstone, Honggou Fm (44-40 Ma)	38.7°N, 101.04°W	2
HY-11-04	93	Fluvial sandstone, Xieja-Chetougou Fm (25.5-18 Ma)	39.7°N, 101.04°W	2
DZ-11-04	92	Fluvial sandstone, Qijiachuan Fm 50-44 Ma)	36.4°N, 102.03°W	2
**QUATERNARY LOESS DEPOSITS – CHINESE LOESS PLATEAU**				
HL1	84	Loess, Heimugou section	35.725883°N, 109.06631°W	3
HL9	165	Loess, Heimugou section	35.725883°N, 109.06631°W	3
HL15	239	Loess, Heimugou section	35.725883°N, 109.06631°W	3
HL33	39	Loess, Heimugou section	35.725883°N, 109.06631°W	3
XF-L1	90	Loess, Xifeng section	35.883°N, 107.966°W	4
10YG-1	96	Loess, Weinan section	34.35°N, 109.516°W	4
ljg	131	Loess, Xifeng section	35.78°N, 107.78°W	5
LGM Loess	84	Loess, Huanxian section	36.6224°N, 107.2867°W	6
CH11-04-01	124	Loess, Jingbian section	37.498717°N, 108.90468°W	7
CH11-04-05	122	Loess, Jingbian section	37.498717°N, 108.90468°W	7
CH11-04-09	76	Loess, Jingbian section	37.498717°N, 108.90468°W	7
CH11-04-11	97	Loess, Jingbian section	37.498717°N, 108.90468°W	7
CH11-05-03	102	Loess, Beiguoyuan section	36.622583°N, 107.28672°W	7
CH11-05-06	107	Loess, Beiguoyuan section	36.622583°N, 107.28672°W	7
CH11-05-10	83	Loess, Beiguoyuan section	36.622583°N, 107.28672°W	7
CH11-05-11	121	Loess, Beiguoyuan section	36.622583°N, 107.28672°W	7
CH11-05-12	76	Loess, Beiguoyuan section	36.622583°N, 107.28672°W	7
CH11-06-01	104	Loess, Lingtai section	34.987367°N, 107.55223°W	7
CH11-06-02	84	Loess, Lingtai section	35.987367°N, 107.55223°W	7
CH11-06-03	105	Loess, Lingtai section	36.987367°N, 107.55223°W	7
CH11-06-04	76	Loess, Lingtai section	37.987367°N, 107.55223°W	7
CH11-06-05	80	Loess, Lingtai section	38.987367°N, 107.55223°W	7
**NORTHERN DESERTS**				
13DUNE01	94	Aeolian dune, Central Ordos Basin	38.98389°N, 108.16321°W	8
13COSMO01	84	Top of Yardang (Mesozoic sandstone)	38.9746°N, 107.3386°W	8
13COSMO02	92	Top of Yardang (Mesozoic sandstone)	38.972°N, 107.343°W	8
13COSMO03	90	Top of Yardang (Mesozoic sandstone)	39.1011°N, 107.8649°W	8
13DUNE03	81	Aeolian dune, Tengger Desert	40.07233°N, 103.92398°W	8
13DUNE05	88	Aeolian dune, Tengger Desert	39.28238°N, 102.69492°W	8
13DUNE06	91	Aeolian dune, Tengger Desert	38.80672°N, 102.36845°W	8
MD10	98	Aeolian dune, Western Mu Us	37.9239°N, 107.991°W	9
MD09	108	Aeolian dune, Western Mu Us	38.4923°N, 107.227°W	9
MD08	119	Mesozoic aeolian sandstone, eastern Mu Us	39.573°N, 108.5151°W	9
MD07	93	Aeolian dune, eastern Mu Us	38.6528°N, 109.6644°W	9
MD06	58	Mesozoic aeolian sandstone, eastern Mu Us	37.992°N, 108.87°W	9
MD05	115	Quaternary fluvial deposits, eastern Mu Us	37.9871°N, 108.822°W	9
MD04	74	Aeolian dune, Western Mu Us	37.706°N, 108.488°W	9
MD03	113	Quaternary sand dune, western Mu Us	37.722°N, 108.488°W	9
MD02	117	Aeolian dune, eastern Mu Us	38.477°N, 108.763°W	9
MD01	152	Aeolian dune, eastern Mu Us	38.136°N, 109.791°W	6
TD1	86	Aeolian dune, Tengger Desert	38.589°N, 105.478°W	6
**QAIDAM BASIN - PALEOGENE FLUVIAL DEPOSITS**				
11HTG16DZ	396	Fluvial sandstone, Shangganchaigou Fm., 35.2-22 Ma	38.25545°N, 90.86796°W	This paper
HTG-E	98	Fluvial sandstone, Xiaganchaigou Fm., 43.8-37.8 Ma	38.333333°N, 90.916667°W	10
GS-E	52	Fluvial sandstone, Xiaganchaigou Fm., 43.8-37.8 Ma	37.5°N, 92.166667°W	10
STB2	93	Fluvial sandstone, Shangganchaigou Fm., 35.2-22 Ma	37.5°N, 92.166667°W	10
Q123	91	Fluvial sandstone, Xiaganchaigou Fm., 43.8-37.8 Ma	37.75°N, 91°W	10
Q7	93	Fluvial sandstone, Xiaganchaigou Fm., 43.8-37.8 Ma	37.75°N, 91°W	10
Z2	69	Fluvial sandstone, Shangganchaigou Fm., 35.2-22 Ma	37.75°N, 91°W	10

NORTHEAST TIBET – PALEOGENE FLUVIAL DEPOSITS				
H6	99	Fluvial sandstone, Xunhua Basin, 28 Ma	36.1°N, 102.4°W	11
H1	98	Fluvial sandstone, Xunhua Basin, 24.5 Ma	36.1°N, 102.4°W	11
H2	95	Fluvial sandstone, Xunhua Basin, 22.5 Ma	36.1°N, 102.4°W	11
WGS17	97	Fluvial sandstone, Linxia Basin, 25 Ma	35.8°N, 103.17°W	11
WGS16	99	Fluvial sandstone, Linxia Basin, 21.5 Ma	35.8°N, 103.17°W	11
WGS15	96	Fluvial sandstone, Linxia Basin, 14.5 Ma	35.8°N, 103.17°W	11
S080946	80	Fluvial sandstone, Guide Basin, Xining Group (ca. 52-21 Ma)	35.05°N, 101.4°W	12
S080911	84	Fluvial sandstone, Guide Basin, Xining Group (ca. 52-21 Ma)	36.258°N, 101.583°W	12
S080950	66	Fluvial sandstone, Guide Basin, Lower (?) Guide Group (ca. 21-19	35.87°N, 101.583°W	12
S080920	54	Fluvial sandstone, Guide Basin, Lower Guide Group (ca. 21-19 Ma	36.083°N, 101.65°W	12

Supplementary Table 1. ***Sample compilations used in the study***.

	Best fit to red mudstones	
	For the minimum dissimilarity value δ_{min}	For the average dissimilarity value Δ_{min}
fluvial supply (Xining Paleogene sandstones)	76 %	68 %
Paleogene Qaidam Baisn	20 %	24 %
Paleogene northeast Tibet	0 %	8 %
Northern deserts	4 %	0 %

Supplementary Table 2. ***Combinations yielding minimum dissimilarity value δ_{min} and lowest average dissimilarity value Δ_{min}.*** *δ_{min} and Δ_{min} are alternative ways to quantify the source mixture that best fits the red mudstone age distribution[8]. δ_{min} is the minimum dissimilarity value among the N synthetic age distributions and for the whole array of potential source combinations. The combinations that best fit the red mudstones can be seen as those for which this minimum value is reached for at least one of their N synthetic distributions. Alternatively, Δ_{min} is the average dissimilarity value among the N synthetic age distributions per potential source combination. The combinations that best fits the red mudstones can be seen as those for which Δ_{min} is the lowest.*

Supplementary References:

1. Botev, Z.I., Grotowski, J.F. & Kroese, D.P. 2010. Kernel density estimation via diffusion. *The Annals of Statistics* 38, 2916-2957.

2. Zhang, J., Wang, Y., Zhang, B., & Zhang, Y. 2015. Tectonics of the Xining Basin in NW China and its implications for the evolution of the NE Qinghai□Tibetan Plateau. *Basin Research*, in press.

3. Pullen, A., Kapp, P., McCallister, A., Chang, H., Gehrels, G., Garzione, C., Heermance, R. & Ding, L. 2011. Quaidam Basin and northern Tibetan Plateau as dust sources for the Chinese Loess Plateau and paleoclimatic implications. *Geology* 39, 1031-1034.

4. Xiao, G., Zong, K., Li, G.; Hu, Z., Dupont-Nivet, G., Peng, S. & Zhang, K. 2012. Spatial and glacial-interglacial variations in provenance of the Chinese Loess Plateau. *Geophysical Research Letters* 39, L20715.

5. Che, X. & Li, G. 2013. Binary sources of loess on the Chinese Loess Plateau revealed by U–Pb ages of zircon. *Quaternary Research* 80, 545-551.

6. Stevens, T., Palk, C., Carter, A., Lu, H. & Clift, P. 2010. Assessing the provenance of loess and desert sediments in northern China using U-Pb dating and morphology of detrital zircons. *GSA Bulletin* 122, 1331-1344.

7. Bird, A., Stevens, T., Rittner, M., Vermeesch, P., Carter, A., Andò, S., Garzanti, E., Lu, H., Nie, J., Zeng, L., Zhang, H. & Xu, Z. 2015. Quaternary dust source variation across the Chinese Loess Plateau. *Palaeogeography, Palaeoclimatology, Palaeoecology* 435, 254-264.

8. Licht, A., Pullen, A., Kapp, P., Abell, J. & Giesler, N. 2016. Aeolian cannibalism: reworked loess as the main sediment source of the Chinese Loess Plateau. *GSA Bulletin*, B31375, doi: 10.1130/B31375.1.

9. Stevens, T., Carter, A., Watson, T., Vermeesch, P., Ando, S., Bird, A., Lu, H., Garzanti, E., Cottam, M. & Sevastjanova, I. 2013. Genetic linkage between the Yellow River, the Mu Us desert and the Chinese Loess Plateau. *Quaternary Science Reviews* 78, 355-368.

10. Cheng, F., Fu, S., Jolivet, M., Zhang, C., & Guo, Z. 2015. Source to sink relation between the Eastern Kunlun Range and the Qaidam Basin, northern Tibetan Plateau, during the Cenozoic. *Geological Society of America Bulletin*, B31260-1, in press.

11. Lease, R., Burbank, D., Hough, B., Wang, Z. & Yuan, D. 2012. Pulsed Miocene range growth in northeastern Tibet: Insights from Xunhua Basin magnetostratigraphy and provenance. *GSA Bulletin* 124, 657-677.

12. Liu, S., Zhang, G., Pan, F., Zhang, H., Wang, P., Wang, K. & Wang, Y. 2013. Timing of Xunhua and Guide basin development and growth of the northeastern Tibetan Plateau, China. *Basin Research* 25, 74-96.
